# High-dose regimens of hypomethylating agents promote transfusion independence in IPSS lower-risk myelodysplastic syndromes: a meta-analysis of prospective studies

**DOI:** 10.18632/aging.202767

**Published:** 2021-03-26

**Authors:** Ziqi Wan, Bing Han

**Affiliations:** 1 Department of Hematology, Peking Union Medical College Hospital, Chinese Academy of Medical Science, China

**Keywords:** azacytidine, decitabine, lower-risk myelodysplastic syndromes, efficacy, side effects

## Abstract

The hypomethylating agents (HMAs) azacytidine (AZA) and decitabine (DAC) are usually administered after the failure of erythropoietin-stimulating agents for lower-risk myelodysplastic syndromes (LR-MDS). However, it is unclear whether one of these HMAs has superior efficacy and safety. This was investigated in the present study by means of a meta-analysis of prospective studies published between January 1990 and July 2020 in PubMed, EMBASE, CENTRAL, and ClinicalTrials.gov databases; 19 studies with 1076 patients were included in the final analysis. The transfusion independence (TI) rate (66.7% [95% confidence interval: 41.7%–87.4%]) was higher with AZA 75 mg/m^2^/day for 7 days than with other regimens (all p<0.025). The proportion of patients with intermediate-1 risk influenced overall survival (p<0.05). There were no differences in treatment response, survival, and adverse event rates between patients treated with AZA (75 mg/m^2^/day for 5 days) and DAC (20 mg/m^2^/day for 3 days), although the latter group had a higher rate of grade 3/4 anemia (15.8% vs 0.0%; p<0.0001) and lower rate of diarrhea/constipation (6.9% vs 25.0%; p=0.002). Thus, both HMAs at high doses achieved reasonable response and TI rates with acceptable side effects, but did not prolong the overall survival in LR-MDS patients.

## INTRODUCTION

Myelodysplastic syndromes (MDS) are heterogeneous myeloid malignancies that manifest as dysplastic blast cells. From 2001 to 2015, around 4 or 5 individuals per 10^5^ per year were diagnosed with MDS in the United States [1]. The 5-year overall survival (OS) of MDS patients is 31.3%, which is lower than the rate in other cancers such as prostate and breast cancers [1]. Nearly half of deaths were the result of acute myeloid leukemia (AML) transformation and over 80% were directly MDS-related [2]. Based on the percentage of bone marrow (BM) blasts, karyotype, and cytopenia, the International Prognostic Scoring System (IPSS) categorizes MDS into the following 4 risk groups: low, intermediate-1 (int-1), int-2, and high. Lower-risk MDS (LR-MDS) encompasses low and int-1–risk groups and is scored as 0 or 1 [3]. Upon occurrence of transfusion dependence in patients with LR-MDS, treatment with erythropoietin (EPO)-stimulating agents (ESAs) followed by lenalidomide, thrombomimetic agents, and hypomethylating agents (HMAs) is initiated [4]. Lenalidomide is most effective in patients with 5q deletion (5q−) [5]. Thrombopoietin mimetics can potentially reduce bleeding events in patients with thrombocytopenia [6]. HMAs that have shown promising results in the treatment of LR-MDS include azacytidine (AZA) [7, 8] and decitabine (DAC) [9, 10]. A previous systematic review concluded that these 2 HMAs both achieved higher response rates than best supportive care (BSC); however, the outcome of LR-MDS patients was not reported because there were insufficient data [11]. A higher overall response rate has been reported with low-dose DAC compared to low-dose AZA [12], but conflicting results were obtained with different regimens [13, 14]. While AZA has traditionally been administered via the subcutaneous (sc) and intravenous (iv) routes, an oral formulation is being evaluated in a phase 3 trial (NCT01566695). Nonetheless, overall survival (OS) rates remain unsatisfactory, and it is unclear whether some HMAs can achieve better outcomes than others. This was investigated in the present study through a meta-analysis of studies comparing the efficacy and safety of AZA and DAC in the treatment of LR-MDS. We also explored factors potentially influencing treatment response and survival.

## RESULTS

### Study selection

**Figure 1:**
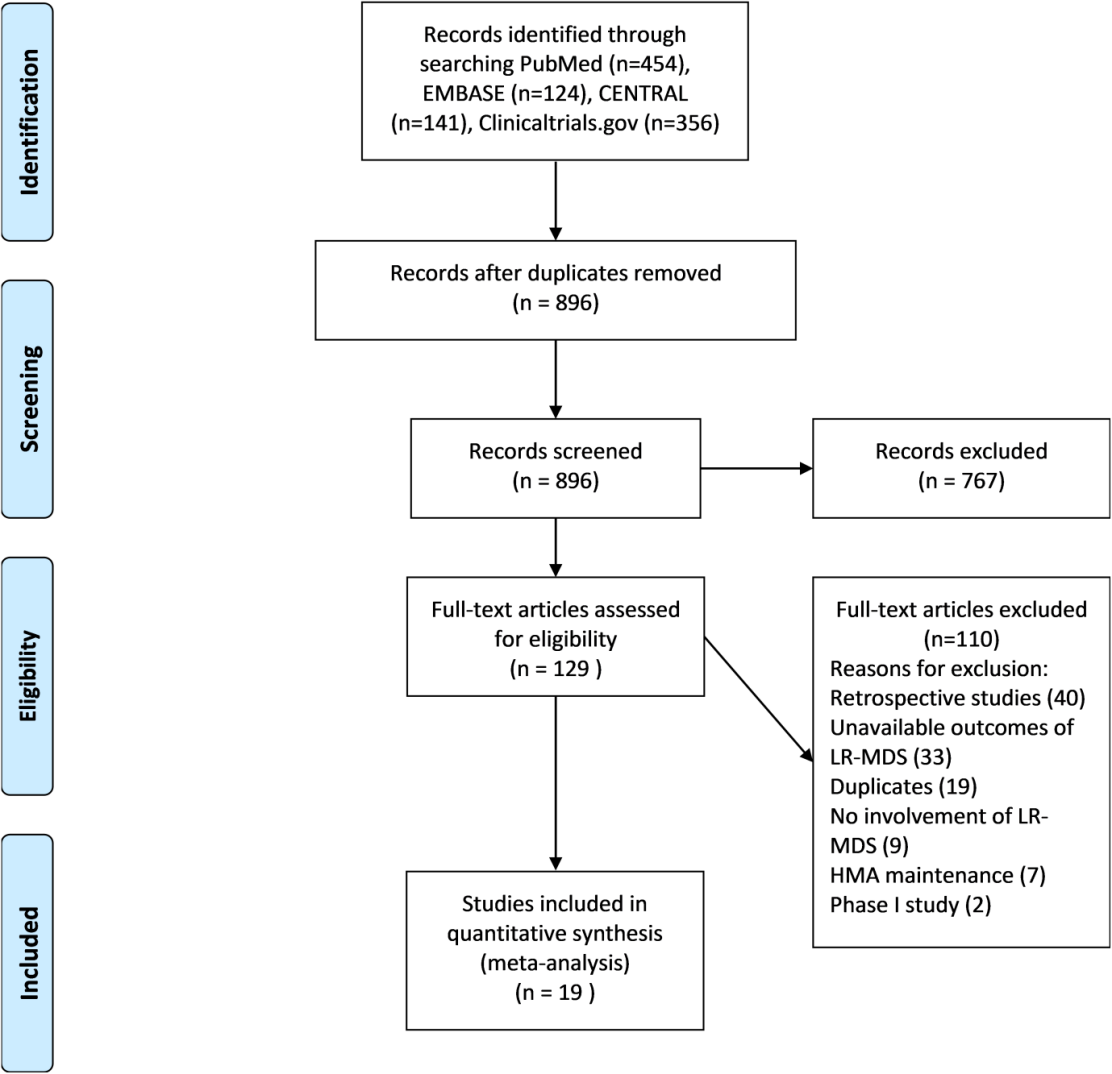
**PRISMA flow chart eligibility criteria of articles included in meta-analysis.** PubMed, EMBASE, CENTRAL and clinicaltrial.gov were selected to search articles between January 1990 and July 2020, using keyword “azacytidine or decitabine and myelodysplastic syndromes”. 1057 items were identified and 179 duplicate studies were removed. The remaining 896 studies were under title and abstract review. A total of 767 articles was excluded due to inappropriate type of study and uninterested intervention. 129 studies were retrieved to assess full text. 19 articles were included in this meta-analysis after depth review.

A total of 896 articles were retrieved from searches of PubMed, EMBASE, CENTRAL, and ClinicalTrials.gov databases; 767 were removed as they were an inappropriate type of publication (eg, retrospective study, review, case report, phase 1 study, or nonhuman study) or evaluated an intervention other than an HMA (eg, lenalidomide, chemotherapy, stem cell transplantation), leaving 129 articles for full-text review. Another 110 articles were excluded for the following reasons: inappropriate type of study (retrospective study, n=40; phase 1 study, n=2); unavailable outcome (n=33); duplication (n=19); unrelated to LR-MDS (n=9); and inappropriate type of intervention (HMA maintenance, n=7). Ultimately, 19 studies were included in the meta-analysis (Figure 1). The characteristics of these studies are shown in Supplementary Table 2.

### Baseline characteristics of patients

A total of 1076 patients were enrolled in the 19 studies, including 177 graded as low (16.4%), 866 as int-1 (80.5%), and 33 (3.1%) with missing classification. Nine studies (47%) reported the baseline characteristics of the 655 participants (Supplementary Table 1). The mean age was 69.2 years (95% confidence interval [CI]: 67.6%–70.7); the proportion of males in 7 studies was 70.1% (95% CI: 66.6%–74.7%); and 11 patients (1.2%; 95% CI: 0.3%–2.6%) had 5q− MDS. Thus, HMAs were mainly used in older, male, int-1–risk patients without 5q deletion.

Of the 425 patients from 8 studies with a clear record of treatment before HMA, 32 (7.5%) were nonresponders to EPO and 160 (37.6%) were refractory/nonresponders to ESA. Additionally, 147 patients (34.6%) were treatment naïve; 43 (10.1%) were treated only with growth factors (ESA and/or granulocyte colony-stimulating factor), and in 43 (10.1%) the treatment was unknown.

For 523 patients in 7 studies, the exact clinical manifestation of MDS was reported; refractory cytopenia with multilineage dysplasia (RCMD) was the most common (n=122, 23.3%), followed by refractory anemia with excess blasts-1 ([RAEB-1]; n=89, 17.0%), refractory anemia with ringed sideroblasts ([RARS]; n=81, 15.5%), refractory anemia ([RA]; n=57, 10.9%), and RCMD with ringed sideroblasts ([RCMD-RS]; n=28, 5.4%). Chronic myelomonocytic leukemia was diagnosed in 32 patients (6.1%), and the incidence of other clinical entities including 5q−, MDS unclassifiable, MDS/myeloproliferative neoplasm unclassifiable, and RAEB-2 was <5%. Nine patients diagnosed as RAEB-2 were graded as LR-MDS and included in our analysis after we verified the original paper. Because of ambiguity in the original data, 17 patients were classified as RCMD or RCMD-RS and 18 were either RA or RARS; 58 cases (11.1%) were not diagnosed as a distinct clinical entity (Supplementary Table 1).

Eight studies with 634 patients reported either the karyotype or IPSS cytogenetic risk; 448 cases were evaluated as good or favorable, 103 as intermediate, and 43 as poor or unfavorable. In 19 patients it was unclear whether there were chromosomal abnormalities and in 21, chromosome information was lacking. The pooled rate of favorable karyotype was 71.7% (95% CI: 65.3%–77.7%). Four studies included gene mutation profiles. Mutations in the following genes were detected in more than 150 patients: *SF3B1* (45.8%; 95% CI: 25.5%–66.9%), *TET2* (33.8%; 95% CI: 27.1%–40.8%), *DNMT3A* 12.2% (95% CI: 5.0%–22.0%), and *ASXL1* (12.1%; 95% CI: 5.5%–20.8%). The mean percentage of BM blasts from 3 studies was 4.0% (95% CI: 3.5%–4.4%). Other parameters such as EPO level, absolute neutrophil count (ANC), red blood cell count, hemoglobin (Hb) level, and ferritin level before treatment were also recorded (Supplementary Table 1).

### Response rate

We conducted univariate analyses to determine whether response rate varied according to the combinations of complete remission (CR), partial remission (PR), marrow (m)CR, and hematologic improvement (HI) used by included studies, and found that different combinations of these parameters did not significantly influence response rate (p=0.46). Thus, the response rates reported in different studies were comparable. Of the 19 studies included in the meta-analysis, 13 used World Health Organization (WHO) criteria and 6 used French–American–British (FAB) criteria for the diagnosis of LR-MDS. International Working Group (IWG) 2006 response criteria were adopted in 15 studies, and the other used IWG 2000 criteria. Only 2 studies evaluated EPO combined with HMA. In univariate analyses, we found no association between diagnostic criteria (WHO or FAB), response criteria (IWG 2006 or 2000), or use of EPO and response rate (all p>0.10; Table 1).

**Table 1:** Univariate meta-regression analysis for response rate, transfusion independence, and overall survival.

		**Different combination**	**Diagnostic criteria**	**IWG criteria**	**EPO usage**	**Different regimens**
Response Rate	p	0.4575	0.3864	0.8537	0.9558	<0.0001
	I^2^	81.57%	81.93%	80.42%	83.62%	20.61%
Transfusion Independence	p	Not applicable	Not applicable	Not applicable	0.1222	0.0034
	I^2^				47.10%	0.00%
Overall Survival (1yr)	p	Not applicable	Not applicable	Not applicable	0.29	0.1609
	I^2^				62.37%	53.16%

I^2^: residual heterogeneity / unaccounted variability.

To identify possible factors influencing the response rate, we compared the age, sex ratio, risk scores, unfavorable cytogenetics, and gene mutation profiles of the patients in the 9 studies. Although slight heterogeneity in age was observed (I^2^=54.2%), this was minimized (I^2^=18.4%) after omitting Thépot’s study [15]. The mean age in the remaining 8 studies was 68.5 years (95% CI: 67.4%–69.7 years). There was no difference in response rates between Thépot’s study and the other 8 studies (p=0.46). Sex ratio did not differ among the 7 studies that included this information (I^2^=0.0%). Given the marked differences in the percentage of int-1–risk patients (I^2^=93.5%) in 17 of the 19 studies, we divided the studies into the following 3 groups based on this parameter: group A (n=8, 95%–100%), group B (n=7, 71%–89%), and group C (n=2, 53%–61%), but found no significant difference in the percentage of int-1–risk patients within each group (I^2^=40.2%, 0.0%, and 0.0%, respectively). Additionally, response rates did not differ among the 3 groups (p=0.93). Thus, age, sex ratio, and proportion of int-1–risk patients did not influence response rates. A slight difference in cytogenetics was found in the 8 studies that reported this data (I^2^=59.4%); we therefore divided the studies into the following 2 groups based on the percentage of patients with a favorable karyotype: group A (n=3, 59%–67%) and group B (n=5, 71%–80%). There were no significant differences in the proportion of patients with favorable karyotype (both I^2^=0.0%) and response rate (p=0.88). The frequency of *TET2* mutation was similar across 4 studies (I^2^=0.0%) but *ASXL1*, *DNMT3A*, and *SF3B1* mutation rates varied (I^2^=59.8%, 74.0%, and 87.4%, respectively). Categorizing the studies based on mutation frequencies had no effect on response rate (all p>0.09).

The therapeutic schedule was found to significantly influence response rate in the univariate analysis (p<0.0001; Table 1). We re-evaluated this parameter according to the following 6 variables: days of administration in 1 treatment cycle (3, 5, 7, 14, or 21); length of 1 cycle (28 or 42 days); mode of administration (sc, iv, oral, or sc/iv); dosage of HMA in each cycle (15 or 20 mg/m^2^/day for DAC, 50 or 75 mg/m^2^/day for AZA, and 300 mg/day for oral AZA); frequency of administration (1 or 3 times per day); and consecutive vs intermittent administration. For example, a regimen of 75 mg/m^2^/day sc AZA for 5 days every 28 days was recorded as 5/28/sc/75/1/consecutive.

The number of days of drug administration, dosage, and interval of administration did not influence the response rate (all p>0.45). However, the route of administration showed a tendency to influence the rate (p=0.07), which was significantly higher with a mixed route of administration (iv/sc) than with other routes. However, as the exact mode of mixed administration was not reported, which could affect the results of our analysis, we examined the pooled response rates with iv, sc, and oral drug administration and found no significant differences between any 2 of these routes (iv: 42% [95% CI: 30%–55%], sc: 35% [95% CI: 29%–41%]; oral: 33% [95% CI: 21%–46%]; all p≥0.30).

The length of 1 treatment cycle and number of doses per day were significantly associated with response rate (both p=0.029). The pooled response rate was 18% (95% CI 8%–30%) for a 42-day, 3 times daily DAC cycle, which was significantly lower than regimens with a 28-day cycle and once-daily administration (46% [95% CI: 38%–53%]; p=0.0003). We compared only regimens with the latter schedule, which included AZA 75 mg/m^2^/day for 7 days (75×7; n=1), 75 mg/m^2^/day for 5 days (75×5; n=5), 75 mg/m^2^/day for 3 days (75×3; n=1), 50 mg/m^2^/day for 6 days (50×6; n=1), and 50 mg/m^2^/day for 5 days (50×5; n=1); and DAC 20 mg/m^2^/day for 5 days (20×5; n=4) and 20 mg/m^2^/day for 3 days (20×3; n=2). The highest response rate was with AZA 75×7 (60.9%), followed by DAC 20×5 (52.5%), AZA 75×3 (48.7%), DAC 20×3 (46.1%), AZA 75×5 (43.5%), AZA 50×6 (42.9%), and AZA 50×5 (38.8%). As most of the regimens were used in just 1 or a few studies with significant heterogeneity, a meta-analysis was not possible. For 2 comparable studies, DAC 20×5 achieved a slightly better response than AZA 75×5, although this result was nonsignificant (p=0.09).

The pooled response rate for the whole cohort was 43.6% (95% CI: 35.5%–51.8%) (forest plot in Supplementary Figure 1). The pooled CR rate from 4 studies was 21.3% (95% CI: 10.0%–35.4%); the pooled mCR rate from 4 studies was 6.4% (95% CI: 2.2%–12.7%); the pooled PR rate from 3 studies was 4.3% (95% CI: 0.0%–20.1%); and the pooled HI rate from 8 studies was 33.7% (95% CI: 17.8%–-51.8%).

### Transfusion independence (TI)

Of the 8 studies reporting TI rate, 1 adopted FAB diagnostic criteria and the others used WHO criteria. Two studies used DAC and the others used AZA. One study used an EPO/HMA combination. All studies used the IWG 2006 response criteria. There were no significant differences in age, sex ratio, and rate of favorable cytogenetics among these studies (I^2^=23.0%, 0.0%, and 34.0%, respectively). Univariate analysis showed that HMA dosage significantly influenced TI rate (p<0.01; Table 1). The therapeutic regimens were AZA 75×7 (n=1), 75×5 (n=3), 75×3 (n=1), and 50×5 (n=1) and DAC 20×3 (n=2). AZA 75×7 achieved a higher TI rate (66.7% [95% CI: 41.7%–87.4%]) than the other regimens (all p<0.025). There was no difference in TI rate between AZA 75×5 and DAC 20×3 (31.5% vs 34.8%; p=0.69); however, both were superior to AZA 50×5 (15.3%; both p<0.017) and tended to be higher than AZA 75×3 (15.8%; p=0.15 and 0.08, respectively).

Given the significant heterogeneity in the proportion of int-1–risk patients (I^2^=78.0%), the studies were categorized as group A (n=1, 100%), group B (n=4, 71%–85%), and group C (n=2, 53%–61%). There were no significant differences in int-1 risk rate within groups, although group A had a higher TI rate than the other 2 groups (p=0.002 and 0.004). For multivariate analysis, therapeutic regimens were divided into 3 groups based on similarities in the TI rate: group 1, AZA 75×5; group 2, AZA 75×5 and DAC 20×3; and group 3, AZA 50×5 and AZA 75×3. Regimens with different dosages showed significant differences in TI rate (group 1 vs 2, p=0.04; group 1 vs 3, p<0.0001; group 2 vs 3, p=0.006), whereas the proportion of int-1–risk patients had no effect on TI rate (p=0.65). The pooled TI rate from 8 studies with 299 patients was 30.5% (95% CI: 21.4%–40.5%) (forest plot in Supplementary Figure 2).

### Survival

Ten studies reported OS including 4 for AZA, 5 for DAC, and 1 for both. One study investigated an EPO/HMA combination. Three studies diagnosed patients according to FAB criteria and the others used WHO criteria. All studies followed the IWG 2006 response criteria. There were no significant differences in age, sex ratio, and rate of favorable cytogenetics (I^2^=28.5%, 0.0%, and 44.6% respectively) across groups, indicating that these factors did not influence OS. A univariate analysis demonstrated that only IPSS score and type of HMA significantly influenced 1- and 2-year OS (p=0.0005 and 0.01, respectively). Given the significant heterogeneity in the proportion of int-1–risk patients, studies were divided into the following 3 groups: group A (n=4, 98%–100%), group B (n=3, 71%–85%), and group C (n=2, 53%–61% after removing 1 study that did not report the exact percentage of int-1–risk patients). There was no significant difference in the proportion of int-1–risk patients across groups (all I^2^=0.0%). Group C had longer 1- and 2-year OS (92.8% [95% CI: 87.8%–96.6%] and 80.2% [95% CI: 72.9%–86.6%], respectively) than group B (86.3% [95% CI: 81.3%–90.6%], p=0.05 and 69.1% [95% CI: 61.9%–75.9%], p=0.03, respectively); group B had longer 1- and 2-year OS than group A (77.4% [95% CI: 70.9%–83.2%], p=0.02 and 51.6% [95% CI: 42.8%–60.3%], p=0.003, respectively); and 1- and 2-year survival rates were longer with AZA than with DAC (1 year: 92.4% [95% CI: 85.7%–97.1%] vs 81.4% [95% CI: 76.5%–85.9%], p=0.009; 2 years: 77.4% [95% CI: 70.5%–83.6%] vs 59.2% [95% CI: 48.9%–69.1%]), p=0.003). The multivariate analysis showed that only the proportion of int-1–risk patients significantly influenced OS (group A vs group B, p=0.03; group A vs group C, p=0.01). The pooled 1-year OS from 10 studies was 86.5% (95% CI: 81.2%–91.0%) and the 2-year OS from 9 studies was 69.5% (95% CI: 61.3%–77.1%) (forest plots in Supplementary Figures 3, 4).

### Adverse events (AEs)

AEs were reported by 7 studies that did not differ in terms of mean patient age (I^2^=9.9%), sex ratio (I^2^=0.0%), or proportion of patients with favorable cytogenetics (I^2^=49.0%) or int-1–risk (I^2^=34.2%). The most frequent grade 3/4 hematologic AE was neutropenia (24.0% [95% CI: 15.9%–33.2%]), followed by anemia (15.9% [95% CI: 12.2%–19.9%]) and thrombocytopenia (11.5% [95% CI: 8.4%–15.1%]). Pancytopenia occurred in 1.2% of patients. The route of drug administration had no effect on the rate of hematologic AEs (p>0.10). As oral administration increased the rate of gastrointestinal AEs, which was also observed in our analysis (p<0.0001), a study evaluating oral AZA [16] was excluded from the calculation of nonhematologic (NH)AE rate. NHAEs pooled from at least 2 studies included nausea at a rate of 12.9% (95% CI: 8.7%–17.8%), fatigue at 11.1% (95% CI 6.9%–16.1%), diarrhea/constipation at 13.3% (95% CI: 4.2%–26.5%), and fever at 8.2% (95% CI: 4.6%–12.8%).

### Subgroup analysis of DAC and AZA

To better compare the efficacy of DAC and AZA and eliminate the possible influence of baseline factors, we analyzed studies that were comparable in terms of patients’ baseline characteristics after removing those that included only int-1–risk patients, did not specify risk category, or showed significant variations in terms of mean age, sex ratio, and proportion of 5q− patients. Four studies were ultimately selected for the subgroup analysis including 2 studies for AZA [17, 18], 1 for DAC [19], and 1 for both [12]. There were no significant differences in age, sex ratio, or rate of 5q− or int-1 risk between patients treated with DAC vs AZA (p>0.45; Table 2). Most of the studies in the subgroup used either AZA 75×5 or DAC 20×3 over a 28-day cycle. The study in which oral AZA was used was not included in the subgroup analysis.

**Table 2:** Subgroup analysis of DAC and AZA.

	**AZA**	**DAC**	**p**
[95%CI]			
IPSS risk: low rate	0.2615 [0.0998; 0.4667]	0.2527 [0.1840; 0.3283]	0.9319
Mean Age (years)	69.5556 [67.4896; 71.6215]	68.5057 [66.4060; 70.6054]	0.4848
Gender: male rate	0.6936 [0.5740; 0.8012]	0.7600 [0.3853; 0.9850]	0.7201
5q-	0.0193 [0.0000; 0.0739]	0.0172 [0.0000; 0.0824]	0.9436
			
Response	0.4221 [0.3090; 0.5394]	0.3849 [0.0938; 0.7326]	0.844
TI^1^	0.2599 [0.1607; 0.3735]	0.3480 [0.2386; 0.4662]	0.274
OS^2^			
1-year	0.8545 [0.7798; 0.9158]	0.8642 [0.8022; 0.9160]	0.8308
2-year	0.6962 [0.6039; 0.7812]	0.6948 [0.5989; 0.7828]	0.982

^1^TI, transfusion independence; ^2^OS, overall survival.

The pooled response rate for patients treated with AZA (n=101) was 42.2% (95% CI: 36.0%–53.9%), which did not differ from that of patients treated with DAC (n=135; 38.5% [95% CI: 9.4%–73.3%]) (p=0.84). There was also no significant difference in TI rate between the 2 groups (AZA: 26.0% [95% CI: 16.1%–37.4%]; DAC: 34.8% [95% CI: 23.9%–46.6%]; p=0.27) (Table 2).

AZA was not superior to DAC in pooled 1- and 2-year OS in the subgroup analysis (1 year: 85% [95% CI: 78%–92%] vs 86% [95% CI: 80%–92%]; 2 years: 70% [95% CI: 60%–78%] vs 69% [95% CI: 60%–78%]; both p>0.80) (Table 2).

There were no differences between AZA and DAC groups in the rates of grade 3/4 neutropenia (32.2% vs 31.7%, p=0.96), grade 3/4 thrombocytopenia (18.8% vs 23.7%, p=0.66), nausea (13.9% vs 12.2%, p=0.74), fatigue (10.0% vs 11.4%, p=0.80), and fever (9.3% vs 6.9%, p=0.56). However, grade 3/4 anemia occurred at a higher rate in patients treated with DAC as compared to AZA (15.8% vs 0.0%, p<0.0001), whereas the opposite was observed for diarrhea/constipation (6.9% vs 25.0%, p=0.002).

### Sensitivity analysis and publication bias

Omitting any study included in the analysis of response rate and OS did not significantly reduce their overall heterogeneity. There was no significant asymmetry in the funnel plots of the 19 studies (p>0.05).

## DISCUSSION

This meta-analysis was carried out in order to compare the efficacy and safety of 2 HMAs, AZA and DAC, in the treatment of LR-MDS. The baseline characteristics of the patients were similar to those that have been previously reported in trials of DAC or AZA alone [20–22]. The participants had a mean age of 69 years, with no opportunity for stem cell transplantation. Around 70% were male, 90% were diagnosed as 5q− MDS, and 80.5% were int-1–risk patients. Based on these features, the patients were potential candidates for HMA treatment.

After eliminating the potential influence of WHO/FAB classification, IWG 2000/IWG 2006 criteria, and inclusion of EPO in the therapeutic regimen, we ruled out the effect of different drug combinations on response rate. Furthermore, we found that age, sex ratio, and IPSS risk did not influence response rate. We did not directly compare the response rates between low- and int-1–risk MDS because of a lack of detailed data. Instead, we classified the studies into 3 groups according to the proportion of int-1–risk patients but found no differences in response rates between groups, which indicated that IPSS risk was not associated with response rate in the present cohort with a high percentage of int-1–risk LR-MDS patients, in agreement with earlier reports [15, 19].

Response rates were similar between patients differing in terms of rate of favorable karyotype or gene mutation profile. A screen of 42 genes commonly mutated in MDS failed to identify significant predictors of treatment response [18], which was confirmed by other studies examining the association between gene mutations and response rate in LR-MDS patients [12, 15, 17]. It has been demonstrated that higher-risk MDS with chromosome 7 abnormality can benefit from AZA treatment [23]. Mutations in the *TET2*, *DNMT3A*, and *TP53* genes were shown to predict a good therapeutic response to HMAs [24–27], whereas *SRSF2*, *U2AF1*, and *ZRSR2* mutations were unrelated to DAC treatment outcome [28]. As LR-MDS patients have much lower rates of unfavorable karyotype or *TET2*, *DNMT3A*, and *TP53* mutations, their treatment response is less likely to be influenced by cytogenetic or molecular abnormalities than that of their higher-risk counterparts.

After eliminating possible interfering factors, the pooled overall response rate was 43.6%, with 21.3% CR, 6.4% mCR, 4.3% PR, and 33.7% HI. Most of the patients in this cohort were either treatment naïve or refractory only to ESA/EPO. HMAs may be a reasonable treatment option for these patients, although they should be used with caution given their high toxicity and long-term AEs. The patients in our study were mainly ESA/EPO-refractory and transfusion-dependent, and could have a very poor outcome if left untreated. Based on the clinical characteristics of our cohort, HMAs could benefit relatively high-risk patients (int-1–risk) within the LR-MDS group, at least for the short term, as suggested by other studies [20, 21]. However, because of the paucity of studies and lack of detailed information, we were unable to determine which WHO/FAB classifications would benefit most from HMAs TI rate is an important parameter for LR-MDS patients as it reflects patients’ quality of life and risk of leukemic transformation and mortality. The pooled TI rate in our study was 30.5%, which is higher than that achieved by most treatments after failure on ESAs [29]. Interestingly, this rate was similar to that of newly reported agents such as luspatercept (38% in a phase 3 trial) [30] and imetelstat (37% in a phase 2 trial) [31], which are costly and not available in many parts of the world.

The meta-analysis revealed that the therapeutic regimen influences treatment response rate. DAC 15 mg/m^2^/8 h for 3 days every 42 days was inferior to the other regimens, which is consistent with a previous report demonstrating that it was less effective than DAC 20×5 every 28 days for all-risk MDS [21]. However, among 28-day regimens, AZA 75×7 and DAC 20×5 had the highest response rates, although a statistical analysis was not possible because of the high heterogeneity, or did not show statistically significant differences because of the limited data. Accordingly, AZA 75×7 had a higher TI rate than other regimens. Thus, AZA or DAC at higher doses (eg, AZA 75×7 or DAC 20×5) may be most effective for the treatment of LR-MDS in terms of both response and TI rates. The latter gradually declined with decreasing dose: lower TI rates were observed with DAC 20×3 and AZA 75×5 than with AZA 75×7, which was nonetheless superior to AZA 50×5. This is supported by other studies demonstrating that the standard AZA 75×7 regimen had superior efficacy to the relatively lower-dose 75×5 regimen [32, 33]. Thus, AZA 75×7 is the recommended HMA for the treatment of LR-MDS. However, different therapeutic regimens did not improve OS, which may be a limitation of this class of drugs. In particular, treatment with HMAs did not prolong OS in patients with low/int-1–risk MDS relative to patients with comparable IPSS scores who received BSC only (1-year OS: 80%–90%; 2-year OS: 65%–85%) [3]. Regarding factors that potentially influence OS, only the proportion of int-1–risk patients impacted 1- and 2-year OS, which is in line with previous findings [3, 34]. In prospective clinical trials, AZA was superior to BSC in prolonging OS [7, 35], but these studies included patients with all risk scores; the effect may be limited to higher-risk patients, as our analyses—which included only lower-risk patients—did not demonstrate a long-term survival benefit with either DAC or AZA.

The AEs of higher-dose regiments warrant consideration because the myelosuppressive effects of HMAs can accumulate with increasing doses. A lack of data hindered our analysis of AEs in patients with LR-MDS treated with AZA 75×7, a regimen that is widely used for AML [36–38] and higher-risk MDS [39, 40] that is associated with a higher rate of hematologic AEs than what was shown by our pooled data. Thus, although higher-dose regimens can achieve better response or TI, AEs would potentially occur more frequently than with lower doses.

We compared the effects of DAC and AZA in patients with comparable baseline characteristics before HMA treatment. However, we were only able to analyze data for 3-day DAC and 5-day AZA regimens because of a lack of suitable publications. Contrary to a previous prospective study that reported a better treatment response with DAC than with AZA [12], we found no significant differences in response or TI rate or OS between the 2 HMAs. The study by Jabbour et al. [12] used a 3-day AZA dosing schedule instead of the widely used 5- or 7-day schedule. As a higher dosage of HMAs can increase response rate, higher dosages of DAC or AZA should be compared in terms of efficacy, although any benefits must be weighed against toxicities. For example, patients treated with DAC had a higher rate of grade 3/4 anemia but a lower rate of diarrhea/constipation in our analysis.

There were some limitations to our meta-analysis. Most of the included studies enrolled patients with int-1–risk LR-MDS and there were limited data on low-risk patients who did not respond to standard treatments. We were also unable to analyze patients with different WHO/FAB classifications because of a lack of detailed information in the original publications. HMA regimens were unbalanced and not all parameters (eg, response and TI rates and OS) were reported. Finally, we excluded some well-designed studies because we were unable to obtain critical data from the publication or from the authors.

## CONCLUSIONS

The results of this meta-analysis demonstrate the efficacy and safety of HMAs in the treatment of LR-MDS in a relatively large cohort. HMAs achieved reasonable response and TI rates with acceptable AE rates, but did not prolong OS compared to BSC. Higher-dose regimens (AZA 75×7 or DAC 20×5) may lead to better clinical outcomes, but the benefits must be balanced with the risk of AEs.

## MATERIALS AND METHODS

This study was carried out according to Preferred Reporting Items for Systematic Reviews and Meta-Analyses guidelines [41].

### Database search

We searched PubMed, EMBASE, CENTRAL, and ClinicalTrials.gov databases for prospective studies conducted between January 1990 and July 2020 using the terms (decitabine OR azacytidine) AND (MDS or myelodysplastic syndromes). Two reviewers (Z.W. and B.H.) independently performed the searches and evaluated and extracted data from the studies; any disagreements were resolved through discussion.

### Inclusion criteria

Studies were included if they were 1) prospective studies (phase 2 or 3); 2) involved IPSS LR-MDS participants—ie, IPSS low and int-1–risk groups, or IPSS score ≤1; 3) evaluated DAC or AZA without other drugs, except in combination with EPO; 4) there was no concomitant stem cell transplantation; 5) reported response rates of LR-MDS patients; and 6) published between January 1990 and July 2020. Retrospective studies, case reports, meta-analyses, commentaries, and reviews were excluded. We included trials comparing the following: DAC or AZA vs placebo or no treatment or BSC; DAC vs AZA; and different dosages or schedules of DAC or AZA. We excluded studies if outcomes of LR-MDS patients were not provided in the article or by the authors.

### Data extraction

The basic characteristics recorded for each study included the first author, year of publication, HMA, treatment schedule, trial number, phase or type of clinical trial, diagnostic criteria for LR-MDS, ethnicity of patients, and response criteria. Additionally, we recorded the baseline characteristics of the study participants including mean age, number of males, number of transfusion-dependent patients, number of 5q− patients, number of BM blast cells, prior treatment, karyotype, gene mutations, median EPO level, median ANC, median Hb, median platelet, median ferritin level, and use of ESA. We also extracted treatment-related data including overall response and TI rates, OS, and AEs if available.

The response rate was defined as the rate of different combinations of CR, mCR, PR, and HI according to IWG 2000 or 2006 criteria. For LR-MDS, TI rate was calculated as the number of TI patients at the time of evaluation relative to the total number of transfusion-dependent patients at baseline [42]. As response and TI rates showed a binomial distribution, they were subjected to arcsine transformation to normalize the proportions. For studies reporting OS, data were extracted either directly from the text or from Kaplan–Meier survival curves using Engauge Digitizer v11.1 for Windows software (https://markummitchell.github.io/engauge-digitizer/). Predicted OS rates at 1 and 2 years were estimated by pooling the OS. If the selected study included both high-risk and LR-MDS data, we used only the latter. We excluded studies that did no report risk categories. A subgroup analysis was conducted to compare the efficacy and safety of AZA and DAC in relation to patients’ baseline characteristics.

### Statistical analysis

The mean age of patients was pooled and the standard deviation estimated from the reported median value and range (or interquartile range) [43, 44]. The pooled rates of males; 5q− MDS; favorable cytogenetics; and *ASXL1*, *DNMT3A*, *TET2*, and *SF3B1* mutation were calculated from available data.

Heterogeneity was considered significant if the I^2^ statistic was >50% or the p value of Cochrane’s Q test was <0.05. A random-effects model was used in the case of significant heterogeneity; otherwise, a fixed-effects model was used. For categorical data such as diagnostic criteria (WHO or FAB), univariate meta-regression analyses were directly carried out to assess their influence on response and TI rates and OS. Single parameters such as age with significant heterogeneity were first categorized into 2 or 3 groups; after ensuring that there was no significant difference within groups, we compared response and TI rates and OS among the groups. Factors showing significant differences in the univariate analyses were used in multivariate analyses.

Publication bias was evaluated by funnel plots and linear regression of Egger’s tests. If the p value was <0.05, the publication bias was considered significant. All statistical analyses were performed using R v3.6.3 for Windows (https://www.r-project.org/).

### Availability of data and materials

Data sharing not applicable to this article as no data sets were generated or analyzed during the current study.

## Supplementary Material

Supplementary Figures

Supplementary Table 1

Supplementary Table 2
