# Supplementary material for: High-dose regimens of hypomethylating agents promote transfusion independence in IPSS lower-risk myelodysplastic syndromes: a meta-analysis of prospective studies
Source: Aging (Albany NY). 2021 Mar 26;13(8):11120–34. doi: 10.18632/aging.202767 (PMC8109092; doi:10.18632/aging.202767)
Supplement: Supplementary Figures [file aging-13-202767-s001.pdf]

SUPPLEMENTARY FIGURES

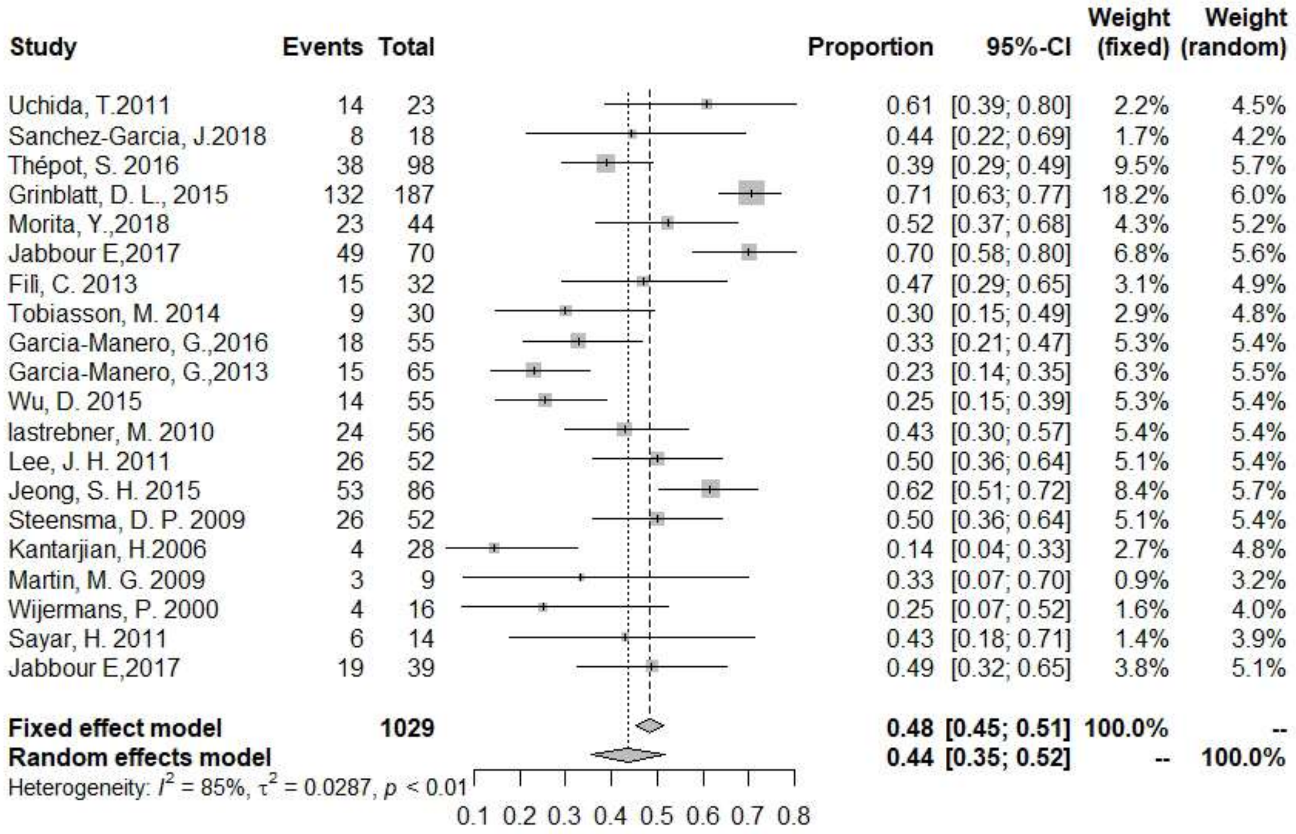

Supplementary Figure 1. Forest plot of response rate among 19 studies included. The pooled response rate for the whole cohort was 43.6% [95%CI 35.5-51.8%].

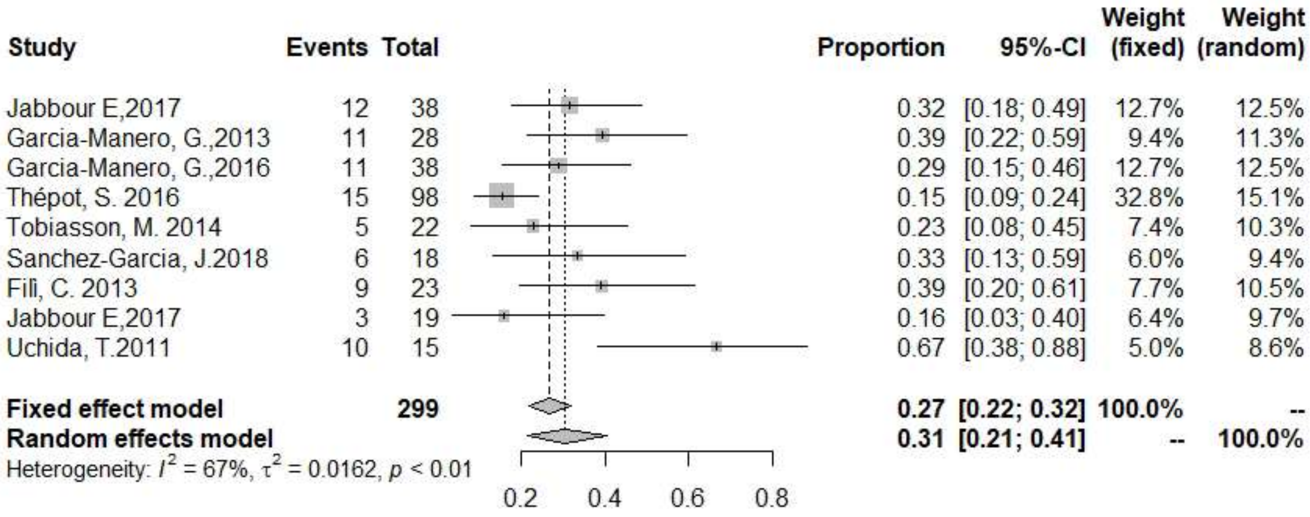

Supplementary Figure 2. Forest plot of transfusion independence rate among 8 studies included. The pooled TI rate was 30.5% [95%CI 21.4-40.5%].

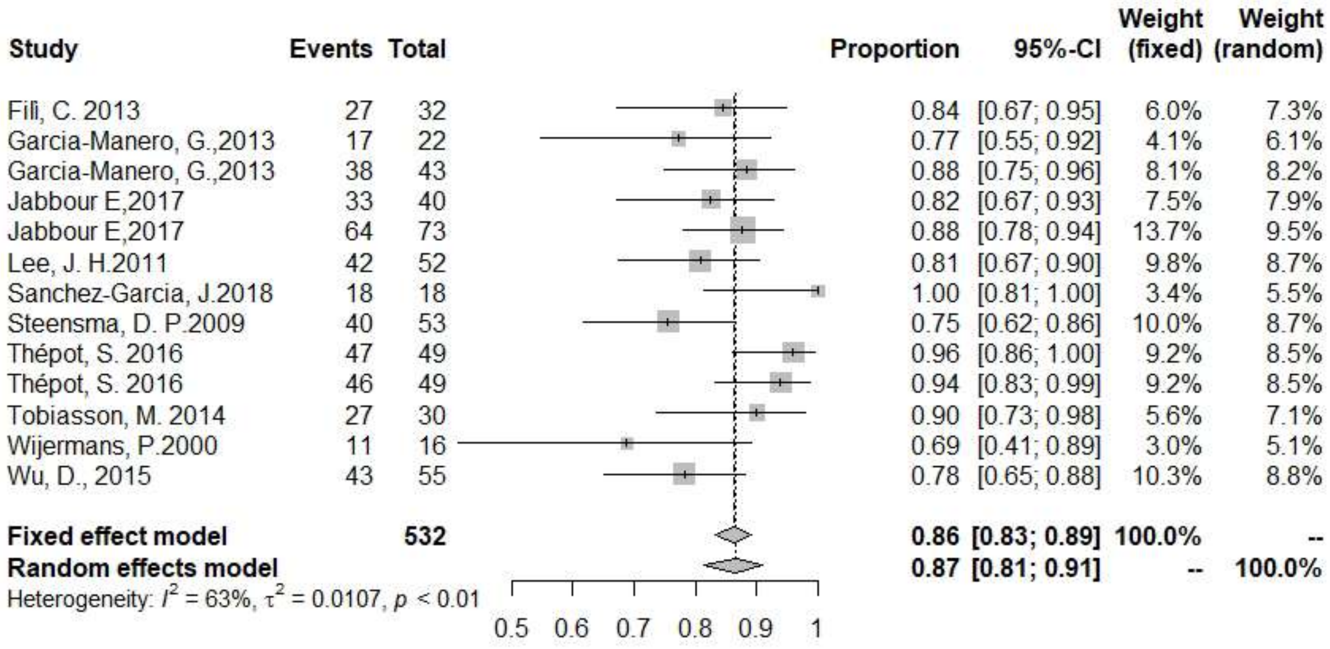

Supplementary Figure 3. Forest plot of 1-yr overall survival. The pooled 1-yr OS from 10 studies was 86.5% [95%CI 81.2-91.0%].

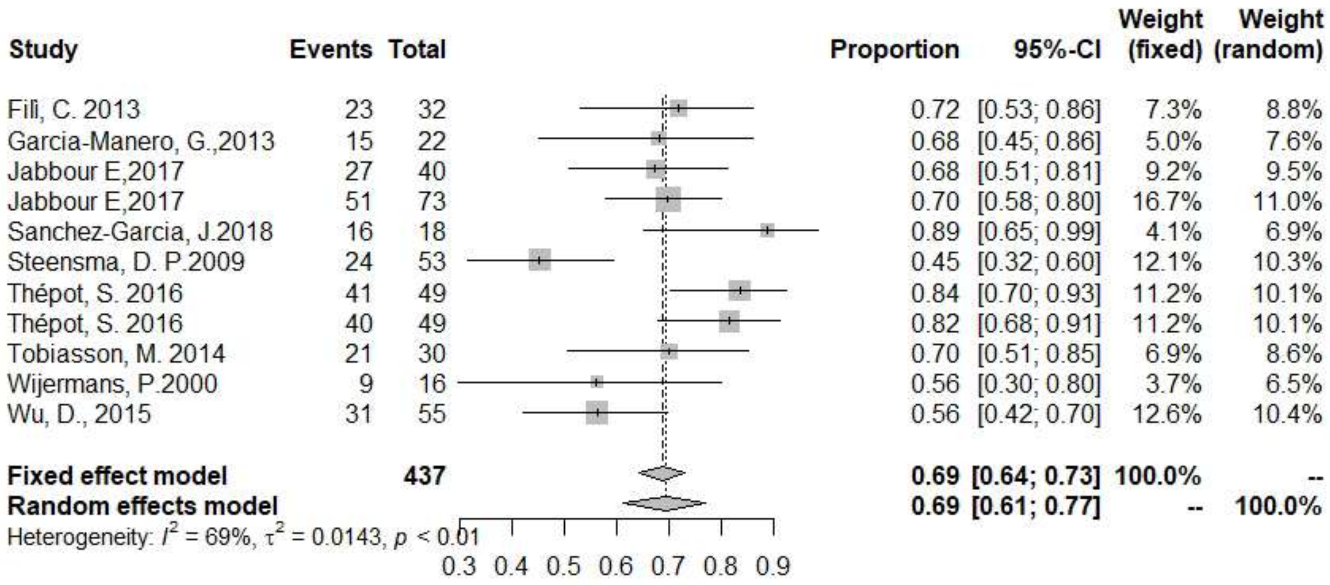

Supplementary Figure 4. Forest plot of 2-yr overall survival. The pooled 2-yr OS from 9 studies was 69.5% [95%CI 61.3-77.1%].
